# Marek’s Disease Virus Infection Induced Mitochondria Changes in Chickens

**DOI:** 10.3390/ijms20133150

**Published:** 2019-06-27

**Authors:** Qin Chu, Yi Ding, Wentao Cai, Lei Liu, Huanmin Zhang, Jiuzhou Song

**Affiliations:** 1 Institute of Animal Husbandry and Veterinary Medicine, Beijing Academy of Agriculture and Forestry Sciences, Beijing 100094, China; 2 Department of Animal and Avian Sciences, University of Maryland, College Park, MD 20740, USA; 3 USDA, Agriculture Research Service, Avian Disease and Oncology Laboratory, East Lansing, MI 48823, USA

**Keywords:** Marek’s disease virus, mitochondrial DNA copy number, gene expression, immune response, T cell transformation

## Abstract

Mitochondria are crucial cellular organelles in eukaryotes and participate in many cell processes including immune response, growth development, and tumorigenesis. Marek’s disease (MD), caused by an avian alpha-herpesvirus Marek’s disease virus (MDV), is characterized with lymphomas and immunosuppression. In this research, we hypothesize that mitochondria may play roles in response to MDV infection. To test it, mitochondrial DNA (mtDNA) abundance and gene expression in immune organs were examined in two well-defined and highly inbred lines of chickens, the MD-susceptible line 7_2_ and the MD-resistant line 6_3_. We found that mitochondrial DNA contents decreased significantly at the transformation phase in spleen of the MD-susceptible line 7_2_ birds in contrast to the MD-resistant line 6_3_. The mtDNA-genes and the nucleus-genes relevant to mtDNA maintenance and transcription, however, were significantly up-regulated. Interestingly, we found that *POLG2* might play a potential role that led to the imbalance of mtDNA copy number and gene expression alteration. MDV infection induced imbalance of mitochondrial contents and gene expression, demonstrating the indispensability of mitochondria in virus-induced cell transformation and subsequent lymphoma formation, such as MD development in chicken. This is the first report on relationship between virus infection and mitochondria in chicken, which provides important insights into the understanding on pathogenesis and tumorigenesis due to viral infection.

## 1. Introduction

Mitochondria, the well-known cytoplasmic organelles for energy making in eukaryotic cells, play important roles in many cell processes, such as small molecules metabolism [1], ion homeostasis [2], immune response [3,4], cell proliferation, and apoptosis [5,6]. Mitochondrion is very special because it contains its own genome (mtDNA), which is a circular molecule and encodes a total of 13 proteins that are all core components of oxidative phosphorylation. There may be hundreds of mitochondria in one cell and one mitochondrion may have multiple copies of mtDNA. It is speculated that the copy number of mtDNA plays a part in mitochondrial biogenesis and regulates mitochondrial functions. Diploid cells may contain a range of 1–10,000 mtDNA molecules depending on cell types and can change over time, where cells with greater energy needs usually have more mitochondria or mtDNA than cells with less needs [7,8]. The change in mtDNA contents is reported to be a useful clinical biomarker for disease diagnose [9,10]. 

Mitochondria are very essential in immune response because they not only involve in the immune and inflammation pathways, but also regulate the activation, proliferation, and function of leukocytes, including macrophages, B and T cells [11,12,13]. Due to the multifunctional characteristics, mitochondria usually serve as the targets of pathogens including viruses [14]. Viral infection can either activate or inhibit mitochondrial functions, alter mitochondrial contents, and influence gene expressions [15]. In return, mtDNA contents often negatively correlate with immune pathways [16] and subsequently influence virus infection and proliferation. Moreover, depletion and damages of mtDNA can lead to inflammation and apoptosis, and ultimately trick oncogenesis in host. 

Marek’s disease (MD) is a highly infectious oncogenic disease of chicken, caused by Marek’s disease virus (MDV), an alpha-herpesvirus. MDV is a DNA herpesvirus that integrates into the host genome upon infection [17,18,19], and is characterized by T cell transformation and fatal lymphomas in visceral organs [20,21]. Nowadays, we have known that genetic and epigenetic background of host have significant effects on MD incidence [22,23,24]. Two highly inbred lines of chickens have been developed for more than half a century at the Avian Disease and Oncology Laboratory (ADOL) [25]. One of the inbred lines, the line 6_3_, was selected for resistance to tumors, while the other, the line 7_2_, was selected for susceptibility, which provides valuable and unique models for immunity and tumorigenesis researches. Recently, a long intergenic non-coding RNA, *GALMD3*, was identified being highly expressed post MDV-infection, which might cause mitochondrial dysfunction and lead to MD in chickens [26]. Although a great amount of efforts has been made for deciphering the virus infection and the host response, the link between MDV infection and mitochondria dynamics remains unclear.

To fill the gap about the function and regulation of mitochondria in MD, this study was designed to examine mitochondrial DNA copy number variation and changes in mitochondrial as well as mitochondria-related nuclear gene expression in three immune organs (bursa of Fabricius, thymus, and spleen). To our knowledge, this is the very first study aimed to explore mitochondrial role in viral infection in chickens.

## 2. Results

### 2.1. Mitochondrial DNA Copy Number Variation

**Figure 1:**
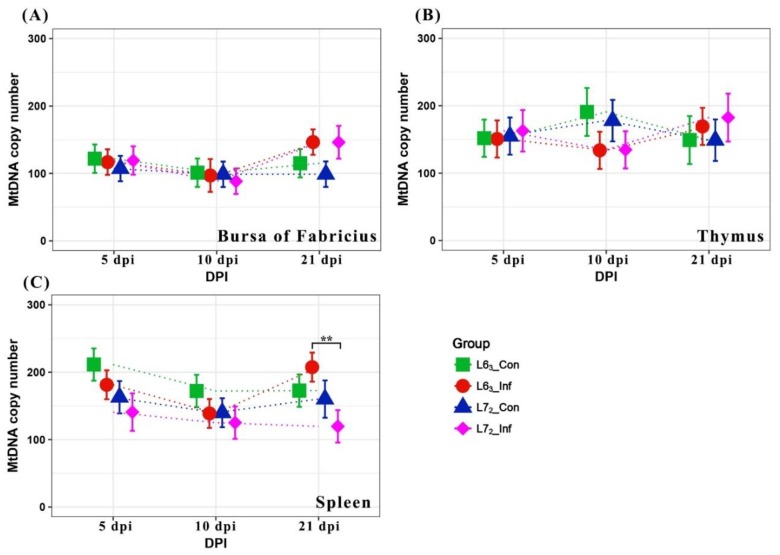
The mitochondrial DNA (mtDNA) copy number variation (means ± standard error) over three time-points. (**A**), (**B**) and (**C**) show the mtDNA abundance in bursa of Fabricius, thymus, and spleen, respectively. MtDNA copies per cell were generated with *ND2* and *β-actin* qPCR data. Birds used in each group were five. The symbols * and ** indicate statistical significance at *p* ≤ 0.05 and *p* ≤ 0.01 levels, respectively, between lines or treatment groups.

The relative content of mtDNA was determined using qPCR analysis of three mitochondrial genes *ND2*, *ND3,* and *COX1*, which encode the NADH dehydrogenase subunit 2 and 3, and the cytochrome C oxidase subunit 1, respectively. The nuclear gene *β-actin* was used as a control. The standard curves showed that the three mitochondrial genes and the *β-actin* control had similar amplification efficiencies, with *ND2* and *β-actin* having the values of 0.90 and 0.95, respectively (Figure S1). Comparison analyses of mtDNA were calculated separately for the three lymphoid organs. The copies of mtDNA per cell predicted with the three mitochondrial genes against the control *β-actin* showed a relative order of *ND3*>*COX1*>*ND2*, though mtDNA variations generated from the three mitochondrial genes exhibited similar trends (see Figure 1 and Figure S2).

The mtDNA copy numbers based on the *ND2* gene over three time-points in the three lymphoid organs are demonstrated in Figure 1. In bursa, the copy numbers of mtDNA remained relatively constant over time in all groups and no difference was observed between the two lines (*p* > 0.05). Likewise, no statistically significant changes in mtDNA contents were detected in thymus (*p* > 0.05). Nevertheless, a significant difference was observed between the two MDV-infected groups at 21 dpi (*p* ≤ 0.01) in spleen, due to a continuously decrease of mtDNA contents in the susceptible birds and an increasing recovery in the resistant birds. The findings implied that 21 dpi was a very important stage for the mitochondria changes after MDV infection. Hence, transcriptome sequencing at this time-point was carried out to further explore the underlying mechanisms.

### 2.2. The Expressions of Mitochondrial DNA-coding Genes

**Figure 2:**
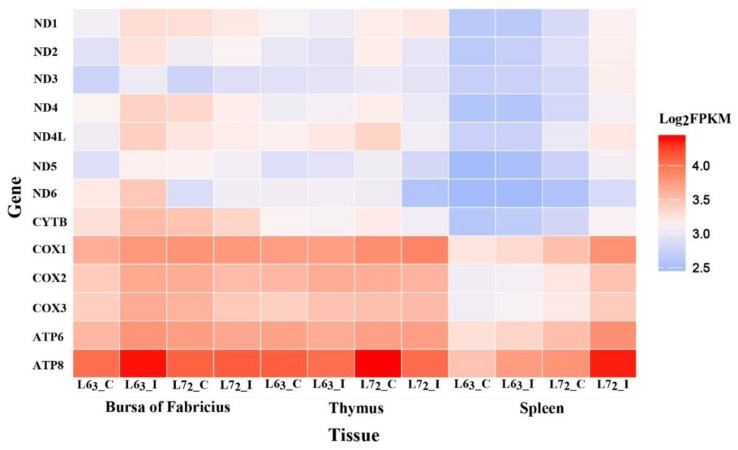
The expression of the 13 mtDNA protein coding genes. The average fragments per kilobase of transcript per million (FPKM) value of the two replicates was used as the gene expression and the heatmap was plotted based on log_2_FPKM. Each row represents an individual gene and each column represents the tissue and group type. The color legend represents the characteristic level, with red indicating high expression level and the blue indicating low expression level. Color density indicates different range of Log_2_FPKM.

To ascertain whether the mitochondrial gene expression was also altered in MD, the 13 mtDNA protein coding genes were examined using RNA sequencing data (Figure 2). Two of the 13 genes, *ATP6* and *ATP8* that encode subunits of the Complex V, showed the higher expression levels, while the genes, *ND1*, *ND2*, *ND3*, *ND4*, *ND4L*, *ND5,* and *ND6* that encode the NADH dehydrogenase (Complex I) subunits, showed the lower expressions. We also found gene expressions were obviously lower in spleen in contrast to those of bursa and thymus. Additionally, the expression levels of the mtDNA genes were noticeably higher in spleen of the line 7_2_ MDV challenged birds than those of the other three groups.

Specific mitochondrial genes with differential expression levels in different comparisons are given in Table 1. After MDV infection, seven and ten out of the 13 mitochondrial genes were up-regulated in spleen of resistant and susceptible lines, respectively. In line 6_3_, the expressions of *ND1*, *ND2*, *ND4*, *ND5*, *COX1*, *COX2*, and *CYTB* were significantly up-regulated with fold changes all between 0 and 1. Besides, *ND3*, *ND6* and *ATP6* were also expressed significantly higher after MDV infection in the line 7_2_ birds than in the controls. All of the ten up-regulated genes in line 7_2_ after infection showed big fold changes. Moreover, the expression levels of eight genes in line 7_2_, such as *ND1*, *ND2*, *ND3*, *ND4*, *ND5*, *CYTB*, *COX2*, and *ATP6,* were higher than those in line 6_3_ (fold changes > 1).

However, no mitochondrial genes changed in bursa of both lines after MDV infection in contrast to the non-infected controls. Uniquely, *ND6* was the only down-regulated gene by MDV challenge in thymus of the line 7_2_ birds.

### 2.3. Differentially Expressed MitoProteome Nuclear Genes

By comparing with the known human and mouse MitoProteome genes (the mitochondrial protein encoding genes), 873 nuclear genes were identified in the chicken genome. Those were further investigated based on the RNA sequencing data and differentially expressed gene (DEG) analysis results performed between lines and treatments.

#### 2.3.1. Mitochondria-Related Nuclear DEGs Induced by MDV

**Figure 3:**
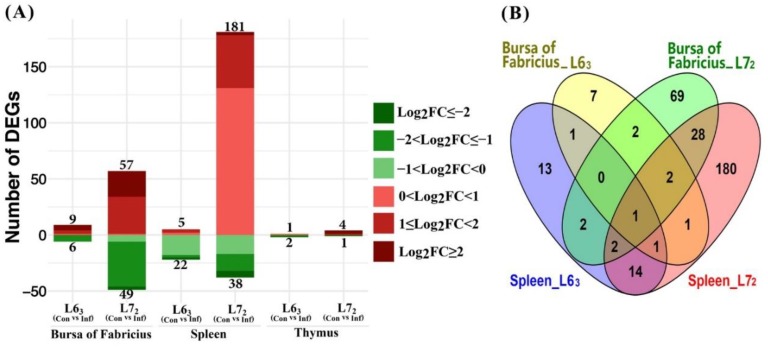
Differentially expressed mitochondria-related nuclear genes in response to Marek’s disease virus (MDV) challenge (FDR ≤ 0.1). (**A**) Number of up- or down-regulated DEGs in three lymphoid organs. Up-regulated and down-regulated genes are displayed in red and green color, separately. Color density indicates different range of Log_2_ (fold change) (log_2_FC). (**B**) Venn diagram showing the number of overlapped DEGs in bursa of Fabricius and spleen from line 6_3_ and 7_2_.

The numbers of differentially expressed nucleus-encoded MitoProteome genes cross the three lymphoid organ tissues between the two lines post MDV challenge are shown in Figure 3. The contrast between MDV-infection and non-infection indicated that MDV challenge induced significantly more DEGs in line 7_2_ than in line 6_3_, and in the line 7_2_ birds most of the DEGs were up-regulated, especially in spleen (Figure 3A). Compared to non-infected control birds, only 27 genes changed in spleen of the line 6_3_ birds, while 219 genes were differentially expressed in spleen of the line 7_2_ birds, which consisted of a quarter (219/873) of all the studied chicken MitoProteome nuclear genes. Among the 219 DEGs observed in spleen of line 7_2_, 181 genes were up-regulated and 38 were down-regulated. In contrast, the number of genes in thymus that changed following infection was very small. Only three DEGs were identified in thymus of the line 6_3_ birds, with the gene *AMN* being up-regulated and *TDRKH* and *SUOX* being down-regulated. Meanwhile, four genes (*STOM*, *OSBPL1A*, *ACOT9* and *C15orf48*) were up-regulated and only one gene *TDRKH* was down-regulated in thymus of the line 7_2_ birds (see Table S1). Interestingly, the *TDRKH* gene was down-regulated by a fold change lower than –2 in both lines. Similarly, the number of significantly changed genes in bursa of line 6_3_ and 7_2_ also had a distinct difference, with the DEG numbers being 15 and 106, respectively.

Since there were only a small number of DEGs identified in thymus, DEG comparison was only performed between the bursa and the spleen (Figure 3B). There were five DEGs in common between the two lines, while 10 and 101 DEGs exclusively between line 6_3_ and line 7_2_ in bursa, respectively. The five common DEGs were *TYSND1*, *ABCB8*, *MRPL17*, *MSRB3*, and *PDK4*. The first three DEGs were up-regulated and the last two were down-regulated in line 6_3_, while all were up-regulated in line 7_2_ (Table S1). However, there were 11 DEGs in common between lines 6_3_ and 7_2_, with 16 and 211 DEGs being exclusively identified in the line 6_3_ and line 7_2_, respectively, in spleen tissues. The 11 common nuclear DEGs were *ABAT*, *COX11*, *MSRB3*, *FKBP10*, *ME3*, *CYP11A1*, *SLC25A30*, *BCL2*, *ACSS3*, *AIFM2*, and *PMP22*. Additionally, we noticed that there were 3 and 24 genes in common between bursa and spleen tissues in the control and infection comparison subsets (L6_3_ vs. L7_2_), respectively. Of note, *MSRB3* was the only gene dysregulated in both bursa and spleen upon MDV challenge in both lines with opposite directions in the two tissues. In bursa the expression of *MSRB3* was up-regulated, while in spleen it was down-regulated.

#### 2.3.2. Mitochondria-Related Nuclear DEGs between Two Chicken Lines

**Figure 4:**
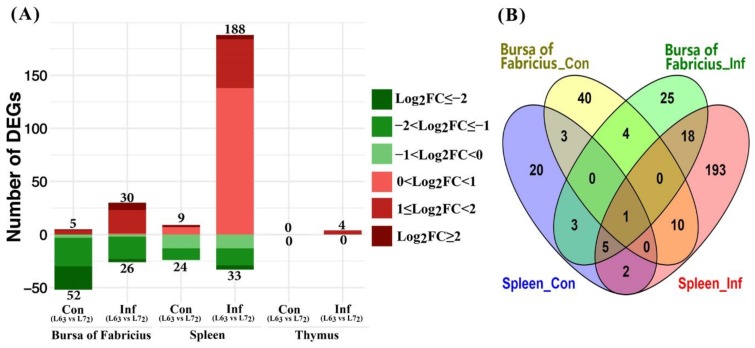
Differentially expressed mitochondria-related nuclear genes between the two lines chickens under each treatment (FDR ≤ 0.1). (**A**) Number of up- or down-regulated DEGs in three lymphoid organs. Up-regulated and down-regulated genes are displayed in red and green color, separately. Color density indicates different range of Log_2_fold change (log_2_FC). (**B**) Venn diagram showing the number of overlapped DEGs in bursa of Fabricius and spleen from the control and infected groups.

The contrasts between the two chicken lines (Figure 4A) showed that there was a total of 221 DEGs in spleen of the line 7_2_ challenged birds compared to line 6_3_ ones; 188 of those DEGs were up-regulated. In contrast, more DEGs (24 out of 33) were expressed at lower levels in spleen of the line 7_2_ control birds than the line 6_3_ birds. In spleen, there were eight DEGs in common between the line comparisons (L6_3_ vs. L7_2_) of MDV challenge and control groups (Figure 4B). These genes were *CMPK2*, *HK2*, *C15orf48*, *HEBP1*, *VDAC1*, *STOML1*, *ACSS3,* and *GLDC*. Among which, the *CMPK2* gene in line 7_2_ was over 2-fold up-regulated.

In bursa tissues, 56 and 57 DEGs were identified between the two lines of the MDV challenge groups and the control groups, respectively. Relatively, more DEGs were up-regulated post MDV challenge in line 7_2_ birds in contrast to the line 6_3_ birds. Again, more DEGs from the comparison of control groups expressed at lower levels in the line 7_2_ than line 6_3_. Five genes, *SCCPDH*, *LAP3*, *CHCHD10*, *STOML1*, and *TDRKH*, were in common between the MDV challenged and control groups of bursa tissues.

No DEG was identified in thymus between the lines of control groups. Four DEGs, *STOM, STOML1, ACOT9*, and *C15orf48*, were identified between the lines in the MDV challenged groups of thymus tissues, which were all up-regulated in expression in the line 7_2_ birds in contrast to the line 6_3_. Additionally, we compared DEGs between the bursa and spleen tissues and noticed that 24 DEGs were in common between the two tissues in the infected groups, while only three were, in the control groups.

#### 2.3.3. Canonical Pathways Prediction

To better understand the biological functions of those differentially expressed mitochondria-relevant nuclear genes, DEGs from the bursa and spleen within the four comparisons were submitted to Ingenuity Pathway Analysis (IPA). Because the DEG numbers in thymus were very small, here we didn’t do the IPA analysis for the thymus. IPA predicts the significance of certain pathways with *p*-value and z-score, which reflect the percentage of genes in the database that are in the pathway and whether the pathway is activated or inhibited, respectively.

**Figure 5:**
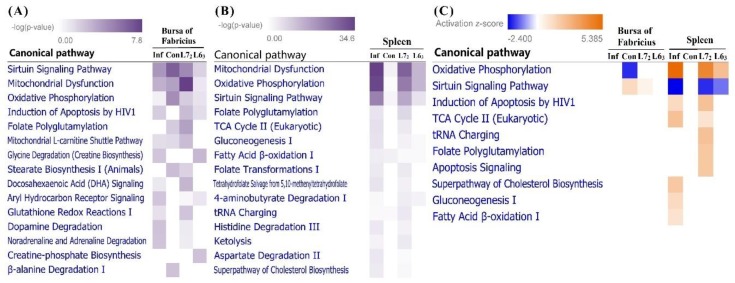
Ingenuity Pathway Analysis (IPA). (**A**) Top 15 canonical pathways based on the DEGs in bursa of Fabricius. (**B**) Top 15 canonical pathways based on the DEGs in spleen. (**C**) The canonical pathways with absolute z-score ≥1 in bursa and spleen. Pathways are sorted by *p*-value or absolute z-score, and the intensity of colors indicates the higher or lower value.

Totally, IPA showed that DEGs in bursa and spleen were significantly enriched in 76 and 101 pathways, respectively (see Tables S2 and S3, *p* ≤ 0.05). After infection, DEGs in bursa of line 6_3_ were enriched in four significant pathways (sirtuin signaling pathway, induction of apoptosis by HIV1, glycine degradation and creatine-phosphate biosynthesis) in contrast with the 51 enriched pathways in line 7_2_. DEGs from spleen of line 6_3_ and 7_2_ in response to MDV infection were significantly enriched in 20 and 66 pathways, respectively. When comparing the top important pathways in bursa and spleen, we found a considerable number of pathways associated with the mitochondrial function and metabolism, such as sirtuin signaling pathway, mitochondrial dysfunction, oxidative phosphorylation (OXPHOS), and folate polyglutamylation (Figure 5A,B).

In bursa, the OXPHOS pathway was significantly inhibited in line 7_2_ control birds compared to line 6_3_ counterparts, while the sirtuin signaling pathway was activated (Figure 5C). Conversely, those two pathways showed opposite regulation directions in spleen, with the OXPHOS pathway being significantly activated and the sirtuin signaling pathway being inhibited in infected birds, especially in line 7_2_. Furthermore, genes involved in the two oppositely regulated pathways were checked. Four genes (*NDUFA2*, *NDUFB2*, *NDUFS5*, and *NDUFV1*), down-regulated in line 7_2_ normal birds than line 6_3_, were shared in the two pathways in bursa. Those four genes encode subunits of mitochondrial complex I (NADH dehydrogenase), which is the first enzyme complex in the respiratory chain. Meanwhile, for the spleen 11 genes were shared in the two pathways, including eight from complex I (*NDUFA8*, *NDUFB1*, *NDUFB3*, *NDUFB4*, *NDUFB5*, *NDUFB8*, *NDUFB9*, and *NDUFS6*), one from complex Ⅱ (*SDHA*), and two from complex V (*ATP5B* and *ATP5C1*).

Very interestingly, we noticed that many genes that encodes the mitochondrial oxidative phosphorylation complexes were up-regulated in spleen of line 7_2_ with MDV infection. When compared to line 6_3_, 28 genes were higher expressed in line 7_2_, including ten from complex I (*NDUFA8*, *NDUFAB1*, *NDUFB1*, *NDUFB3*, *NDUFB*, *NDUFB5*, *NDUFB8*, *NDUFB9*, *NDUFS6*, and *NDUFV3*), two from complex II (*SDHA* and *SDHA2*), five from complex III (*UQCC1*, *UQCRB*, *UQCR10*, *UQCRC1*, and *UQCRH*), six from complex IV (*COX4I1*, *COX5A*, *COX6B1*, *COX7A2*, *COX11*, and *COX17*) as well as five from complex V (*ATP5B*, *ATP5C1*, *ATP10D*, *ATP5G*3, and *ATP5S*).

In addition, several other pathways associated with energy metabolism, such as TCA cycle, gluconeogenesis, and fatty acid β-oxidation, were also activated in susceptible birds, implicating the high-energy demands in this organ.

Meanwhile, two pathways relating with apoptosis (induction of apoptosis by HIV1 and apoptosis signaling) were also activated in spleen of line 7_2_ after infection with MDV, suggesting the non-negligible role of apoptosis in MDV inducing tumorigenesis.

#### 2.3.4. Nuclear Genes Involving in the mtDNA Replication, Transcription and Maintenance

Subsequently, some important nuclear genes, which play essential roles in mtDNA replication, transcription and viability maintenance, were further investigated. No relevant genes were found to be differentially expressed in thymus at 21 dpi while many were found to be dysregulated in the other two lymphoid organs, especially in spleen (Table 2). In bursa, *SLC25A4*, also known as *ANT1*, was the only significantly up-regulated gene in both lines upon MDV infection and the expression of this gene in line 7_2_ was lower than in line 6_3_, whether challenged or not.

In spleen of the MD-susceptible and -resistant lines, infection had contrasting effects on the expression of genes relating to mtDNA replication and transcription. Upon MDV infection, only *POLG2* were up-regulated in line 6_3_, while five genes (*TWNK*, *SSBP1*, *DNA2*, *MGME1*, and *SLC25A4*) showed up-regulation in line 7_2_. Moreover, the expression of eight important genes (*TWNK*, *SSBP1*, *DNA2*, *MGME1*, *SLC25A4*, *TFAM*, *MTERF2*, and *SUPV3L1*) were significantly higher in line 7_2_ infected chickens comparing to the line 6_3_ counterparts, indicating that MDV infection can significantly up-regulate those genes that are closely related to mtDNA replication, transcription, and maintenance. Obviously, the *POLG2* gene expression level was lower in the spleen of line 7_2_ infected birds comparing to line 6_3_, because this gene increased in line 6_3_ while it remained unchanged in line 7_2_.

## 3. Discussion

### 3.1. MtDNA Content and Gene Expression

To our knowledge, this is one of few studies to explore the relationship between mtDNA and avian herpesvirus infection, especially covering the three phases of MDV infection. At 10 dpi, a slight decreasing tendency was observed in thymus with the infection for both lines, indicating that the latency period in thymus deserves further studies. It was reported that herpes simplex virus type 1 (HSV-1) and HSV-2 in human trigger mtDNA damage or loss following by mitochondrial dysfunction and the depleting of mRNA encoded by the mitochondrial genomes [15,27]. Similarly, in our study the mtDNA contents deduced significantly in spleen of the MDV-infected line 7_2_ birds. Interestingly, a significantly elevated mitochondrial gene transcriptional activity was observed. However, mtDNA content alone cannot be used as a surrogate for the respiratory activity in abnormal situations, for example, tumors [28]. At 21 dpi, about 40 percentage cells in spleen were MDV-integrated, whereas only 3.7% cells in thymus were MDV-integrated and bursa had the media number of cells being integrated [18]. Importantly, the virus genome integrating into the host genome is a key feature of tumor cell population [17]. Many studies have implicated that oncoviruses, viruses that transform cells into tumors, can modulate mitochondrial functions and bioenergetics by altering mitochondrial pathways, for example, reprogramming of energy metabolism [29]. Hence, we consider that further studies need to explore the regulatory mechanisms of the transformed cells working with mitochondrial together, which may manipulate cell signaling and energy metabolism of host to fulfill its high-energy demand in virus proliferation phase.

### 3.2. Mitochondria-Related Nuclear Genes and Pathway Analysis

The mitochondrial biogenesis consists of a great deal of proteins. Besides the 13 proteins that encoded by its own genome, there still remain 1000–1500 mitochondrial proteins being encoded by the nuclear genome and imported into mitochondria from the cytoplasm [30]. When comparing the expression of those mitochondria-related nuclear genes in the three immune organs, we found that the thymus had the smallest transcriptional response while the spleen possessed the maximum number of differentially changed genes, which is in consistent with the results from others [31]. As expected, many genes and pathways were altered in spleen of the MD-susceptible birds compared to MD-resistant ones. First, oxidative phosphorylation (OXPHOS), one of the most important function in mitochondria, was significantly activated in spleen of line 7_2_ infected birds. On which, 28 genes included in the mitochondrial oxidative phosphorylation complexes were up-regulated. Besides, several other pathways associated with energy metabolism including gluconeogenesis and fatty acid β-oxidation were also significantly activated in the spleen in line 7_2_. Cancer cells use glucose and glutamine to promote cell growth and proliferation, a process known as metabolic reprogramming [32]. In this process, OXPHOS is essential not only for fulfilling the increased demands for energy to support the high rate of proliferation but also for macromolecules biosynthesis that are critical for enhanced tumor growth [33,34,35]. Coincidentally, the cholesterol biosynthesis pathway, often elevated in proliferating normal tissues and tumors [36], was also activated in line 7_2_. Taken together, we speculate that the transformed lymphocyte in spleen of the MD-susceptible chickens rewired the metabolic process in mitochondria to fulfill the high energy demands.

Another important pathway in MDV infection is sirtuin signaling pathway, which is famous for the roles in metabolism, aging, and cancer [37,38,39]. Sirtuins are nicotinamide adenine dinucleotide (NAD^+)^-dependent deacetylases and can acetylate metabolic proteins, such as tricarboxylic acid (TCA) cycle enzymes, fatty acid oxidation enzymes, and subunits of OXPHOS complexes in response to metabolic stress [40]. Mammals have seven sirtuins, three out of which, SIRT3, SIRT4, and SIRT5, are found to be located in mitochondria [41]. The genes *SIRT3*, *SIRT4*, and *SIRT5* were detected to be dysregulated in bursa and spleen of chickens, illustrating their importance in mitochondrial basic biology upon MDV infection. Additionally, apoptosis signaling is also an import part in spleen upon MDV infection, in which mitochondria play a pivotal role as well. A series of genes responsible for cell programmed death or tumorigenesis were found to be dysregulated, for example, the gene *MSRB3*, *PNPT1*, *AIFM2*, and so on. It is considered that down-regulation of *MSRB3* could increase the levels of cellular reactive oxygen species (ROS) and active intrinsic mitochondrial pathway through increasing the Bax to Bcl-2 ratio and cytochrome c releasing, finally inducing cell apoptosis [5]. Interestingly, the gene *MSRB3* showed conversely regulating styles in bursa and spleen, down-regulated in spleen and up-regulated in bursa. Coincidentally, MDV infection in line 7_2_ increased the expression of *BAK1* (pro-apoptotic) and meanwhile decreased the expression of *BCL2* (anti-apoptotic) in spleen, and meanwhile, the *CYCS* expression was up-regulated, indicating the active apoptosis process in this organ. Another gene *PNPT*1, which was documented recently to be released from mitochondria coordinately with CYCS and to possess a new pro-apoptotic role, was similarly increased in line 7_2_ spleen samples after infection. Additionally, the *AIFM2* gene, a gene with pro-apoptotic function and often being down-regulated in various cancers [42] was indeed significantly decreased in spleen of the line 7_2_ infected birds. Moreover, two mPTP genes *VDAC1* and *VDAC2*, which was reported to be activated by *linc-GALMD3*, an up-regulated long intergenic non-coding RNA in MDV infection leading to apoptosis and cell death [26], were also found significantly up-regulated in spleen samples of the line 7_2_ infected birds. It has been reported that several viruses can induce apoptosis of lymphoid through multiple pathways. MDV replicates in the infected B and T cells may induce apoptosis of various cells including virus-infected cells or transformed cells, resulting in a depletion of lymphocytes and transient immunosuppression in the host [43].

### 3.3. The Mitochondrial DNA Replication and Transcription upon MDV Infection

Although the mitochondria have their own genome, the replication and transcription of mtDNA are completely controlled by the nuclear genome. It is estimated that 250–300 nuclear proteins are dedicated to the replication, transcription, maintenance, and copy number control of this muticopy genome [44]. Many out of the well-appreciated genes, e.g., *TWNK*, *SSBP1*, *DNA2*, *MGME1*, and *SLC25A4* were all detected to be significantly up-regulated in spleen of the line 7_2_ infected birds. TWNK is a mitochondrial 5′–3′ helicase, which binds to and unwinds double stranded DNA and is necessary for replication of mtDNA [45,46]. SSBP1 is the mitochondrial single stranded binding protein, whose function is to restrict initiation of light-strand mtDNA synthesis to the specific origin of light-strand DNA synthesis [47] and SSBP1 also interacts with TWINK and polymerase gamma (Polγ), the only DNA polymerase in mitochondria, to ensure their functions [48]. *DNA2*, *MGME1*, and *SLC25A4* are three genes essential for mitochondrial genome processing, maintenance, and stability, and mutations in those genes are often responsible for the loss of mitochondrial copy numbers [49,50,51,52]. It is also implicated that the high expression of *DNA2* may promote the cancer cells proliferation [53]. Besides, three other important genes *MTERF2*, *SUPV3L1*, and *TFAM* also highly expressed in the line 7_2_ infected birds. The *MTERF2* gene belongs to the mitochondrial transcription termination factor (*MTERF*) family, which has been reported to be linked with the regulation of mtDNA replication and transcription [54,55]. In human *MTERF2* is highly expressed in tissues that are highly dependent on the mitochondrial energy production and may regulate oxidative phosphorylation by modulating mitochondrial DNA transcription [56,57]. The *MTERF* family in mammals has four members, named *MTERF1* to *MTERF4*, in which *MTERF1* was explored more widely. *MTERF1* is considered as a “contrahelicase” in mtDNA replication and may prevent collisions between mtDNA replication and transcription [58]. Thereby, it is possible that *MTERF2* had the similar role and further work is deserved to exploit *MTERF2* functions in chicken. Additionally, mitochondrial helicase SUV3 (encoded by *SUPV3L1*) is predominantly required for the processing of mitochondrial polycistronic transcripts [59]. It is known that SUV3 can interact with polynucleotide phosphorylase (PNPase), that is encoded by *PNPT1*, to form SUV3·PNPase complex and modulate mt-mRNA poly(A) tail lengths in response to changes in energetic states of mitochondria [60], suggesting its crucial role in control of the amount and translation of each mitochondrial mRNA. The protein encoded by the *TFAM* gene is one of the essential components for the mitochondrial DNA transcription, replication, organization, and maintenance [61,62], which can bind, unwind and bend DNA to initiate the mitochondrial transcription. T cells with *TFAM* being depleted proliferated less than wild type T cells [63]. In spleen of the line 7_2_ infected birds, the *TFAM* gene was significantly up-regulated in contrast the counterparts of line 6_3_. Meanwhile, it has been shown that *TFAM* expression is regulated by *PPARGC1A*. Interestingly, the expression of *PPARGC1A* was also up-regulated in spleen of the line 7_2_ infected birds. Combining with the higher expression of mitochondrial genes, it is conceivable that the proliferation of T cells was activated at 21 dpi in spleen of the line 7_2_ birds infected with MDV.

Of note, only the *POLG2* gene in spleen of the MD-resistant line 6_3_ infected birds was significantly up-regulated. *POLG2* encodes the accessory subunits of DNA polymerase gamma (Polγ), which is the only DNA replicative polymerase involved in the human mitochondria and is crucial for the replication and repair of mtDNA [64,65]. Polγ has two subunits: A catalytic subunit and an accessory subunit, which were encoded by *POLG* and *POLG2*, respectively. POLG2 could enhance interactions with the DNA template and increases both the catalytic activity and the processivity of POLG, suggesting it is the major regulator of polymerase activity. In Drosophila melanogaster, over-expression of *POLG2* rather than *POLG* can definitely increase the amount of mtDNA within individual cells [66]. In human, mutations in *POLG2* have a dominant negative effect and lead to multiple mtDNA deletions [67]. In neuronally-differentiated (ND)-PC12 cells being quiescently infected with herpes simplex virus type 1 (HSV-1), *POLG2* was also noted to be down-regulated [68]. Accordingly, we speculate that the upregulation of *POLG2* played a key role in the mtDNA maintenance in line 6_3_.

## 4. Materials and Methods

### 4.1. Ethics Statement

The study protocols for animal experiments were in strict accordance with the Animal Care and Use Committee (ACUC) Guidelines approved by USDA, ADOL (April 2005, Project Number 6040-31320-009-00-D) and the Guide for the Care and Use of Laboratory Animals by Institute for Laboratory Animal Research (Eighth Edition, 2011).

### 4.2. Chickens, Treatment, and Samples

Chicks were obtained from the specified-pathogen-free (SPF) parent flocks of lines 6_3_ and 7_2_, and were housed in a BSL-2 facility on the farm of the Avian Disease and Oncology Laboratory (ADOL, East Lansing, Michigan, USDA). On the fifth day after hatching, young birds were randomly selected and divided into challenge and control groups in each line. The birds of challenge groups for both lines were given a dosage of 500 plaque-forming units (PFU) of 648A passage 40 MDV intra-abdominally each. Chicks of different treatment groups were housed separately in negatively pressured isolators of uniform conditions. Bursa of Fabricius (referred as bursa in this paper), thymus, and spleen samples were collected at 5, 10, and 21 days post-infection (dpi) from 5 birds per line, per group at each of the time-points, which were individually placed in RNAlater (Qiagen, Valencia, CA, USA) immediately and stored at −80 °C until further analysis. Firstly, mtDNA copy number were detected in all 60 samples (3 tissues × 5 individuals × 4 groups). Then, according to the mtDNA variation results, tissues at 21 dpi were decided for gene expression experiment and two individuals were randomly selected from each group.

### 4.3. Quantification of mtDNA Copy Number

Genomic DNA was isolated from all of the sampled tissues using Wizard Genomic DNA Purification Kit (Promega, Madison, WI, USA). The DNA concentration was detected using Synergy HTX Multi-Reader (BioTek, Winooski, VT, USA) and adjusted to 50ng/uL. The relative amounts of mtDNA were determined by qPCR. The *β-actin* gene was used as the reference nuclear gene with the primers: *β-actin*_F GAGAAATTGTGCGTGACATCA, *β-actin*_R CCTGAACCTCTCATTGCCA. Three mitochondrial genes, *ND2*, *ND3*, and *COX1*, were selected in this study, which were described by Reverter et al. [7]. The PCR amplicons were generated on a C1000 Touch™ thermal cycler (BioRad, Hercules, CA, USA) in a 10 μL reaction, which contained 5 μL of 2×SYBR Green PCR mix (Biorad), 3 μL of ddH_2_O, 1 μL primers (10 pmol/μL per primer) and 1 μL DNA template (50 ng/μL). The reactions for each sample were carried out in triplicates along with a negative control (without template). To construct the standard curves, a pooled template was prepared with equal amount of DNA from each of the 30 individual samples (10 for each tissue) and 1:10 dilution series ranging from 1 mg to 0.001 ng were made and used.

Relative mtDNA copy numbers were calculated following equation [7]: MtDNA copy number = 2^1+(Ct_n_gene_−Ct_mt_gene_)^, where Ct represents the average cycle threshold. The mtDNA copy number data from the three tissues were analyzed separately. The PROC GLM procedure in SAS 9.4 was used to carry out the analysis.

### 4.4. RNA Sequencing

Total RNA samples extracted from all three tissues at 21dpi from two randomly selected individuals in each group were used for deep sequencing. The RNA extraction, cDNA synthesis, library preparation, transcriptome sequencing, and qPCR validation were carried out following the reported protocols [69]. Raw RNA-seq data were treated with Trimmomatic first for quality control and mapped onto the chicken reference genome (Gallus gallus Galgal 5.0) using HISAT2. Differential expression analyses were performed with Cuffdiff tools for comparisons between the treatment groups within each line, and also between the lines within each treatment group. Fragments per kilobase of transcript per million (FPKM) was used as the relative gene expression level. Genes showing a *p*-value ≤ 0.05 and a false discovery rate (FDR) ≤ 0.1 were considered differentially expressed genes (DEGs).

### 4.5. MitoProteome Gene Differential Expression Analysis and Pathway Analysis

To fully use the available mitochondrial information, 1158 human MitoProteome genes were downloaded from the website https://www.broadinstitute.org/files/shared/metabolism/mitocarta/human.mitocarta2.0.html, which released the nuclear and mitochondrial DNA genes encoding proteins with strong support of mitochondrial localization [70]. Gene names were directly compared with those in chicken. Finally, 886 genes were matched including 13 mtDNA encoded protein genes as well as 873 mitochondria-related nuclear genes. The gene expression and differential expression data of those MitoProteome genes were picked out from the RNA-seq results for further analysis. The Ingenuity Pathway Analysis (IPA) software was used for gene pathway analysis.

## 5. Conclusions

In summary, in this study we have investigated the variability of mtDNA copy number and gene expression level in the three lymphoid organs in response to MDV challenge. We found that MDV challenge had little impact on mtDNA contents in chickens of the MD-resistant line, but the mitochondrial DNA abundance and gene expression level were obviously altered at the transformation phase, especially in spleen, in chickens of the MD-susceptible line. MDV infection significantly increased the mitochondrial gene expression in the spleen tissue of the MD-susceptible birds, albeit a significant decrease of the mtDNA copy number was observed. Meanwhile, many of the nuclear genes related to mitochondrial genome maintenance and gene expression were up-regulated except for *POLG2*, which was conversely up-regulated in the MD-resistant line. The data indicated that the *POLG2* gene may be a potential regulator for the conflict between the mtDNA copy number and the gene expression of mitochondria in the MD-susceptible birds, directly resulting in imbalance between metabolic and cell signaling and finally the MD pathogenesis and oncogenesis. Further work is warranted to look into mtDNA replication and gene transcription as well as the mitochondria regulation mechanism in relation with MDV infection in chicken.

## Figures and Tables

**Table 1:** Differentially expressed mitochondrial DNA genes in three lymphoid organs.

Tissue	Comparison	Up-Regulated Gene	Down-Regulated Gene
Bursa of Fabricius	Con (L6_3_ vs. L7_2_)		*ND6*
Thymus	L7_2_ (Con vs. Inf)		*ND6*
Spleen	L6_3_ (Con vs. Inf)	*ND1, ND2, ND4, ND5, COX1, COX2, CYTB*	
	L7_2_ (Con vs. Inf)	*ND1, ND2, ND3, ND4, ND5, ND6, COX1, COX2, CYTB, ATP6*	
	Con (L6_3_ vs. L7_2_)		*ND6*
	Inf (L6_3_ vs. L7_2_)	*ND1, ND2, ND3, ND4, ND5, COX2, CYTB, ATP6*	

**Table 2:** Dysregulated nuclear genes involved in the mtDNA replication, transcription, and maintenance.

Tissue	Comparison Subset	Up-Regulated Gene	Down-Regulated Gene
Bursa of Fabricius	L6_3_ (Con vs. Inf)	*SLC25A4*	*POLG2*
	L7_2_ (Con vs. Inf)	*SLC25A4*	*DNA2, MGME1, TK2*
	Con (L6_3_ vs. L7_2_)		*SLC25A4, MPV17*
	Inf (L6_3_ vs. L7_2_)		*SLC25A4, DNA2, TK2*
Spleen	L6_3_ (Con vs. Inf)	*POLG2*	
	L7_2_ (Con vs. Inf)	*TWNK, SSBP1, DNA2, MGME1, SLC25A4*	
	Inf (L6_3_ vs. L7_2_)	*TWNK, SSBP1, DNA2, MGME1, SLC25A4, TFAM, MTERF2, SUPV3L1*	*POLG2*

## Supplementary Materials

Supplementary materials can be found at https://www.mdpi.com/1422-0067/20/13/3150/s1.
