# Supplementary material for: Marek’s Disease Virus Infection Induced Mitochondria Changes in Chickens
Source: Int J Mol Sci. 2019 Jun 27;20(13):3150. doi: 10.3390/ijms20133150 (PMC6651546; doi:10.3390/ijms20133150)
Supplement: Supplementary file 1 [file ijms-20-03150-s001.pdf]

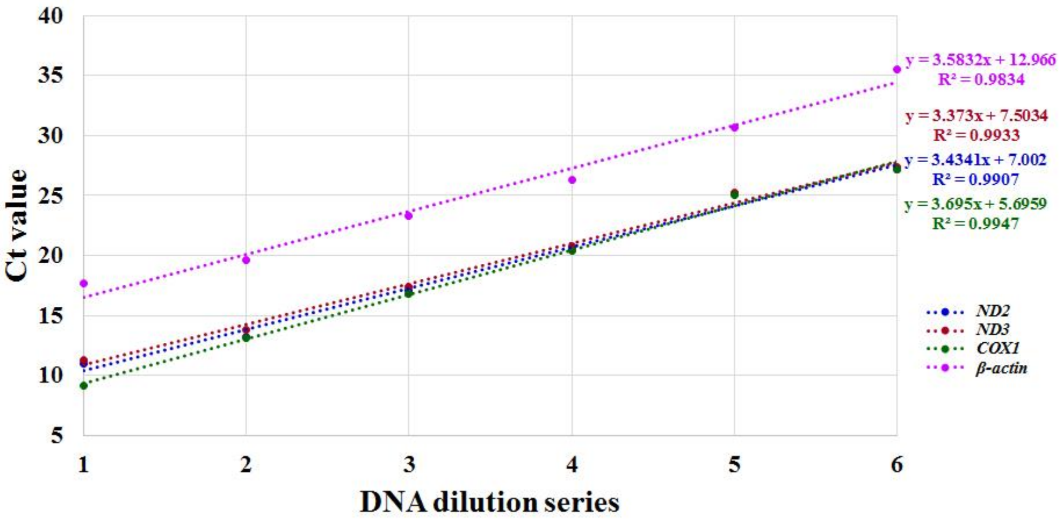

Figure S1. Standard curves to estimate PCR efficiencies for one nuclear gene and four mitochondrial genes. X-axis shows the DNA dilution series, from 1mg to 0.001ng. Y-axis is the cycle threshold (ct) value. The correlation coefficients were from 0.98 to 0.99 and the amplification efficiencies were 0.85 to 0.95.

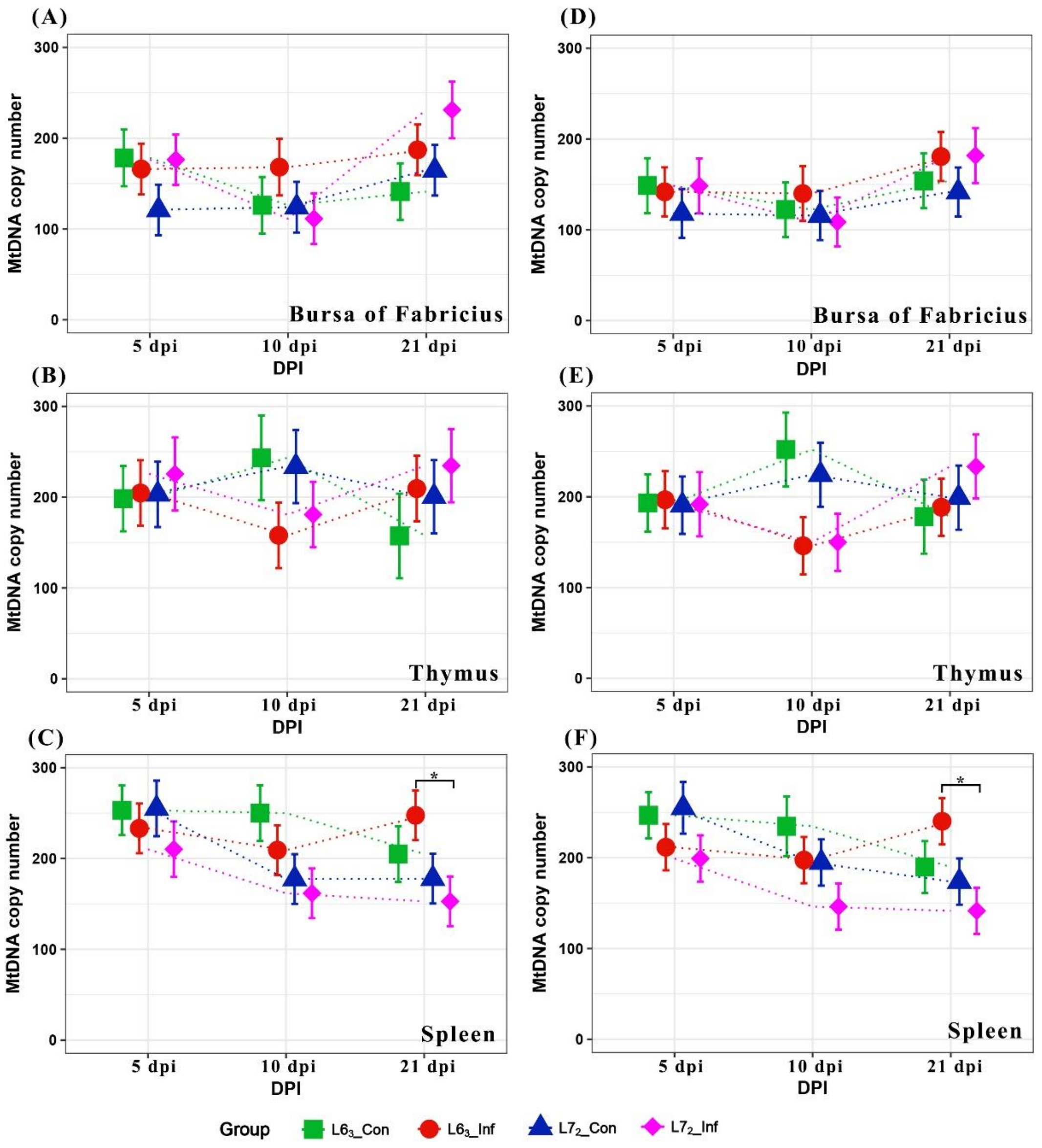

Figure S2. MtDNA copy numbers (means± standard error) predicted with *ND3* and *COX1* against *β-actin*. (A), (B) and (C) shows the variations generated by *ND3* against *β-actin*, and (D), (E) and (F) are the results generated by *COX1* against *β-actin*. Birds used in each group are five. The symbols * and ** mean $p \leq 0.05$ and $p \leq 0.01$, respectively, indicating significant differences between lines or treatments.

Table S1. The differentially up- and down-regulated mitochondria-related nuclear genes among different comparisons in the three lymphoid organs.

Tissue	Comparison subset	No. of DEGs *	No. of up-regulated genes	No. of down-regulated genes	Up-regulated nuclear genes	Down-regulated nuclear genes
Bursa of Fabricius	$L6_3$ (Con vs Inf)	15	9	6	CKMT2, GATM, HSPB7, MSRB3, OBSCN, PDK4, PMP22, SLC25A4, UCP3	ABCB8, MPC2, MRPL17, NDUFB2, POLG2, TYSND1
	$L7_2$ (Con vs Inf)	106	57	49	ABCB6, ABCB8, ACOT7, AGR2, AMT, BCL2, C15orf48, CLPP, CMPK2, COASY, COQ10B, COX14, CPT1A, CYP27A1, ECSIT, EFHD1, EHHADH, EPHX2, FKBP10, FPGS, GCDH, GPD2, GPX1, HEBP1, HINT2, HK2, IDH1, MAOA, MECR, MGST1, MRPL16, MRPL17, MRPL2, MRPL43, MRPL55, MRPS12, MSRB3, NDUFB7, NDUFS5, NDUFV1, NT5DC3, OGG1, OPA3, OSBPL1A, PARS2, PDK4, SLC25A29, SLC25A39, SLC25A44, STOM, STOML1, SUOX, TCIRG1, TIMM10, TOMM6, TYSND1, VDAC1	ACSL4, AKAP1, AKAP10, ATXN2, BCL2L1, BID, CCT7, CHCHD4, CMC2, COMT, COMTD1, DHODH, DHTKD1, DNA2, DUT, FASTKD2, FDPS, FDX1, GDAP1, GLS, GSR, HSPD1, IBA57, IMMT, LAMC1, LONP1, MCCC1, MGME1, MIEF1, MINOS1, MLH1, MMADHC, MRPS10, MTG2, NDUFAF4, NDUFV2, OXCT1, PDK3, PDP2, PGS1, PIF1, PTCD3, RHOT1, SHMT1, TK2, TMEM65, UNG, UQCR11, VDR
	Con ($L6_3$	57	5	52	ABAT, ACSL4, COMTD1, PAK5, PIF1	ABCB8, ACOT9, AGR2, AMT, CHCHD10, CLPP,

	vs $L7_2$)					COA3, COQ10B, COQ4, COX14, CPN2, DDAH1, ECSIT, FASTK, FOXRED1, FPGS, GCDH, GPX1, GPX4, HINT2, LAP3, MPC2, MPV17, MRPL16, MRPL17, MRPL2, MRPL24, MRPL41, MRPL43, MRPL55, MRPS12, MRPS34, NDUFA2, NDUFB2, NDUFB7, NDUFS5, NDUFV1, OGG1, OPA3, PRELID1, ROMO1, SCCPDH, SLC25A34, SLC25A39, SLC25A44, STOML1, SUOX, TDRKH, TIMM10, TIMM13, TOMM6, TYSND1
	Inf ($L6_3$ vs $L7_2$)	56	30	26	ALDH3A2, CPT1A, IDH1, MADD, SIRT3, PC, NDUFAF7, BCL2, EFHD1, MGST1, HK2, VDAC1, TSPO, HEBP1, EPHX2, TXNRD1, SLC25A29, EHHADH, C12orf10, TCIRG1, MECR, MAOA, OSBPL1A, ABCB6, STOM, CYP27A1, NT5DC3, STOML1, C15orf48, CMPK2	OBSCN, GATM, UCP3, HSPB7, TK2, NUDT2, COX7C, SLC25A4, DUT, LAP3, FDPS, TDRKH, COMT, CHCHD10, MRPS31, IBA57, NTHL1, NDUFA4, UNG, SCCPDH, UQCRB, TMEM65, UQCR11, PDK3, DNA2, DBI
Spleen	$L6_3$ (Con vs Inf)	27	5	22	ABAT, COQ9, COX11, POLG2, MAOA	ACSS3, AIFM2, BCL2, C3orf33, CPN2, CYP11A1, FKBP10, GPX1, HINT3, HSD17B4, LAP3, ME3, MRPL41, MRPS36, MSRB3, NDUFS4, NTHL1,

						NUBPL, NUDT2, PMP22, SLC25A30, TDRKH
	$L7_2$ (Con vs Inf)	219	181	38	ABAT, ACAD8, ACAD9, ACAT1, ACLY, ACOT7, ACOT9, ADCK2, AIFM1, ARF5, ATP5C1, ATP5G3, AURKAIP1, BAK1, BRIP1, C12orf10, C15orf48, C1QBP, CCDC51, CCDC58, CCDC90B, CCT7, CHCHD4, CHCHD6, CHDH, CISD1, CISD2, CLPX, COQ3, COX11, COX17, COX6B1, COX7C, CRY1, CS, CYB5R2, CYCS, DBI, DLAT, DLST, DNA2, DNAJC15, DUT, ELAC2, ETFA, FASTKD2, FPGS, GAPDH, GARS, GCDH, GCSH, GOT2, GPAM, GPI, GPX4, GSR, GSTO1, GSTZ1, HADHA, HCCS, HEBP1, HEMK1, HSDL1, HSDL2, HSPA9, HSPD1, IDE, IMMT, LDHB, LONP1, LRPPRC, MADD, METTL8, MGME1, MMAB, MRPL14, MRPL15, MRPL22, MRPL23, MRPL24, MRPL27, MRPL32, MRPL37, MRPL4, MRPL42, MRPL47, MRPL48, MRPL50, MRPL53, MRPL57, MRPL9, MRPS10, MRPS14, MRPS18A, MRPS22, MRPS23, MRPS26, MRPS35, MRPS9, MTERF2, MTFMT, MTHFD1, MTHFD1L, MTIF2, MTX2, NARS2, NDUFA8, NDUFA9, NDUFAB1, NDUFB9, NDUFV3, NT5DC3, OGG1, OXCT1, OXNAD1, PAICS, PARK7, PC, PDE12, PDHA1, PHB, PHB2, PIF1,	AASS, ACO1, ACSS1, ACSS3, AIFM2, AK4, ATP10D, BCL2, CHCHD10, CLIC4, CROT, CYB5A, CYP11A1, DCAKD, DDAH1, ECHDC3, EPHX2, FAM210B, FKBP10, HAGH, IVD, LAMC1, MCEE, MCU, ME3, MSRB3, PDK3, PDK4, PDPR, PMP22, PPM1K, PRSS35, SLC16A7, SLC25A30, SLC25A43, SNAP29, TXNRD1, TYSND1

					PMPCA, PNPT1, PPIF, PRELID1, PRELID3B, PTCD2, PTCD3, PTRH2, PTS, RAB24, RARS2, RMDN1, RTN4IP1, SAMM50, SARS, SDHA, SDHAF2, SFXN5, SHMT1, SIRT4, SLC25A13, SLC25A15, SLC25A17, SLC25A24, SLC25A32, SLC25A38, SLC25A39, SLC25A4, SLC25A47, SOD1, SSBP1, STOM, SUCLG2, SURF1, SYNJ2BP, TAMM41, TARS, TBRG4, TIMM44, TIMM8A, TKT, TMEM126A, TMEM70, TPI1, TRAP1, TRMT10C, TRMT2B, TRMT61B, TSPO, TWNK, UNG, UQCC1, UQCRC1, UQCRC2, UQCRH, USMG5, VDAC2, WARS2	
	Con ($L6_3$ vs $L7_2$)	33	9	24	ABCD2, C15orf48, CMPK2, HEBP1, HK2, HSPB7, PPM1K, STOML1, VDAC1	ACAA2, ACSS3, AGMAT, ALDH7A1, AMACR, ATP5I, COX7C, GLDC, HINT2, HSD17B4, HSDL2, LYRM7, MRPL17, MRPL50, MRPS36, NADK2, NDUFAF2, NDUFS4, NUDT2, PPWD1, SECISBP2, STAR, TARS, THNSL1
	Inf ($L6_3$ vs $L7_2$)	221	188	33	ACAD9, ACAT1, ACOT9, AGK, AK2, ARF5, ATP5B, ATP5C1, ATP5G3, ATP5S, AURKAIP1, BAK1, BCL2L1, BNIP3, C15orf48, C1QBP, CCDC58, CCT7, CHCHD2, CHCHD4, CHCHD6, CHDH,	AASS, ACSF2, ACSS1, ACSS3, AK4, ATP10D, DAH1, DUSP26, ECHDC3, FAM210B, FECH, FKBP10, GLDC, IVD, KIF1B, LAMC1, LETM2,

					CISD1, CISD2, CMPK2, COX11, COX17, COX4I1, COX5A, COX6B1, COX7A2, CPOX, CS, CYB5B, CYB5R2, CYCS, DBI, DECR1, DHCR24, DHRSX, DLST, DNA2, DNAJC15, DNM1L, DUT, ECI1, ECI2, ETFA, FASTKD2, FDPS, FPGS, GAPDH, GARS, GCSH, GFER, GOT2, GPI, GRPEL1, GSR, GSTO1, GSTZ1, GTPBP10, GTPBP3, HADHA, HADHB, HEBP1, HK2, HSPA9, HSPD1, IDE, IMMT, ISCU, LDHB, LONP1, LRPPRC, MADD, MDH1, MDH2, MECR, METTL8, MGME1, MGST1, MIPEP, MLH1, MRPL14, MRPL15, MRPL23, MRPL24, MRPL3, MRPL37, MRPL38, MRPL4, MRPL42, MRPL46, MRPL47, MRPL48, MRPL53, MRPS14, MRPS18A, MRPS22, MRPS23, MRPS25, MRPS26, MRPS28, MRPS31, MRPS33, MRPS35, MRPS5, MRPS9, MTHFD1, MTHFD1L, MTHFD2, MTIF2, MTX2, NDUFA8, NDUFAB1, NDUFB1, NDUFB3, NDUFB4, NDUFB5, NDUFB8, NDUFB9, NDUFS6, NDUFV3, NT5DC3, NUBPL, OGG1, OSBPL1A, PAICS, PAK5, PARK7, PDE12, PDHA1, PHB, PHB2, PIF1, PRELID1, PRELID3B, PSMA6, PTCD3, PTS, PYCR1, RAB24, SAMM50, SARS, SDHA, SDHAF2, SFXN5, SIRT5, SLC25A11,	MAOA, MCU, ME3, MSRB3, NUDT19, PDPR, PMP22, POLG2, PRSS35, SARDH, SERHL2, SFXN3, SLC25A25, SLC25A43, TXNRD1, ZADH2

					SLC25A13, SLC25A15, SLC25A17, SLC25A38, SLC25A39, SLC25A4, SLC25A47, SOD1, SSBP1, STOM, STOML1, SUCLG2, SUPV3L1, SYNJ2BP, TCIRG1, TFAM, TIMM13, TIMM44, TIMM8A, TIMMDC1, TKT, TMEM126A, TMEM70, TOMM22, TPI1, TRAP1, TRMT10C, TRMT11, TSPO, TWNK, UNG, UQCC1, UQCR10, UQCRB, UQCRC1, UQCRH, VDAC1, VDAC2	
Thymus	$L6_3$ (Con vs Inf)	3	1	2	AMN	SUOX, TDRKH
	$L7_2$ (Con vs Inf)	5	4	1	ACOT9, C15orf48, OSBPL1A, STOM	TDRKH
	Con ($L6_3$ vs $L7_2$)	0	0	0		
	Inf ($L6_3$ vs $L7_2$)	4	4	0	ACOT9, C15orf48, STOM, STOML1	

* Genes showing a false discovery rate (FDR)≤0.1 were considered differentially expressed genes (DEGs).

Table S2. The canonical pathways and p value predicted with Ingenuity Pathway Analysis (IPA) analysis in bursa of Fabricius using the differentially expressed MitoProteome genes.

No.	Canonical Pathway	Bursa_L6$_3$ (Con vs Inf)	Bursa_L7$_2$ (Con vs Inf)	Bursa_Con (L6$_3$ vs L7$_2$)	Bursa_Inf (L6$_3$ vs L7$_2$)
1	Sirtuin Signaling Pathway	1.89	4.83	6.53	4.31
2	Mitochondrial Dysfunction	1.01	7.76	3.84	3.22
3	Oxidative Phosphorylation	1.20	2.08	4.60	0.66
4	Induction of Apoptosis by HIV1	1.44	2.79		2.07
5	Folate Polyglutamylation		3.85	2.11	
6	Mitochondrial L-carnitine Shuttle Pathway		2.73	1.58	1.42
7	Glycine Degradation (Creatine Biosynthesis)	2.92			2.34
8	Fatty Acid β-oxidation I		2.19	1.31	1.15
9	Stearate Biosynthesis I (Animals)		1.92	2.66	
10	L-DOPA Degradation		2.12		2.34
11	Death Receptor Signaling	1.26	1.31		1.73
12	Valine Degradation I		1.18	1.55	1.40
13	Docosahexaenoic Acid (DHA) Signaling		2.99		0.95
14	Aryl Hydrocarbon Receptor Signaling	1.09	0.38		2.40
15	Fatty Acid β-oxidation III (Unsaturated, Odd Number)		1.82		2.04
16	Geranylgeranyldiphosphate Biosynthesis		1.82		2.04
17	Glutathione Redox Reactions I		2.43		1.28
18	Glycine Cleavage Complex		1.65	2.03	
19	Trans, trans-farnesyl Diphosphate Biosynthesis		1.73		1.95
20	Trehalose Degradation II (Trehalase)		1.73		1.95
21	LPS/IL-1 Mediated Inhibition of RXR Function		1.28	0.53	1.86
22	Dopamine Degradation		0.88		2.50
23	Cytotoxic T Lymphocyte-mediated Apoptosis of Target Cells		2.19		1.15
24	Noradrenaline and Adrenaline Degradation		0.85		2.43

25	Salvage Pathways of Pyrimidine Deoxyribonucleotides		1.52		1.75
26	Small Cell Lung Cancer Signaling		2.38		0.76
27	Acyl-CoA Hydrolysis		1.35	1.73	
28	BER pathway		1.35	1.73	
29	GDP-glucose Biosynthesis		1.43		1.65
30	Fatty Acid Activation		1.32	1.69	
31	UDP-N-acetyl-D-galactosamine Biosynthesis II		1.39		1.61
32	Glucose and Glucose-1-phosphate Degradation		1.39		1.61
33	Apoptosis Signaling		2.24		0.71
34	Bile Acid Biosynthesis, Neutral Pathway		1.32		1.54
35	Isoleucine Degradation I		1.29		1.51
36	γ-linolenate Biosynthesis II (Animals)		1.20	1.58	
37	Amyotrophic Lateral Sclerosis Signaling		2.06		0.65
38	Type I Diabetes Mellitus Signaling		2.06		0.65
39	PXR/RXR Activation		0.66		2.02
40	Glutaryl-CoA Degradation		1.23		1.45
41	Superpathway of Geranylgeranyldiphosphate Biosynthesis I (via Mevalonate)		1.20		1.42
42	NRF2-mediated Oxidative Stress Response		1.40		1.13
43	Creatine-phosphate Biosynthesis	2.52			
44	β-alanine Degradation I			2.50	
45	4-aminobutyrate Degradation I			2.33	
46	Myc Mediated Apoptosis Signaling		1.47		0.80
47	Cardiolipin Biosynthesis II		2.12		
48	Glutamine Degradation I		2.12		
49	Glycine Biosynthesis I		2.12		
50	Glutamate Degradation III (via 4-aminobutyrate)			2.11	
51	OX40 Signaling Pathway		1.33		0.73
52	Phenylethylamine Degradation I				2.04

53	TGF-β Signaling		1.31		0.72
54	PEDF Signaling		1.31		0.72
55	Glutathione Redox Reactions II		1.82		
56	Glycerol-3-phosphate Shuttle		1.82		
57	IL-8 Signaling		1.38		0.43
58	Thioredoxin Pathway				1.80
59	CD27 Signaling in Lymphocytes		1.76		
60	D-myo-inositol (3,4,5,6)-tetrakisphosphate Biosynthesis		0.37		1.38
61	D-myo-inositol (1,4,5,6)-Tetrakisphosphate Biosynthesis		0.37		1.38
62	2-ketoglutarate Dehydrogenase Complex		1.73		
63	dTMP De Novo Biosynthesis		1.73		
64	3-phosphoinositide Degradation		0.34		1.30
65	Glycerol Degradation I		1.65		
66	Superpathway of Serine and Glycine Biosynthesis I		1.58		
67	Phenylalanine Degradation IV (Mammalian, via Side Chain)				1.51
68	Folate Transformations I		1.47		
69	Leucine Degradation I		1.47		
70	Citrulline Biosynthesis		1.47		
71	Vitamin-C Transport				1.45
72	Glycine Betaine Degradation		1.43		
73	Ketolysis		1.43		
74	Histamine Degradation				1.38
75	Oxidative Ethanol Degradation III				1.33
76	Fatty Acid α-oxidation				1.31

Bursa_L6_3 (Con vs Inf) represents the comparison between the MDV-infected and control birds in line 6_3.

Bursa_L7_2 (Con vs Inf) represents the comparison between the MDV-infected and control birds in line 7_2.

Bursa_Con (L6_3 vs L7_2) represents the comparison between the line 6_3 and 7_2 control birds.

Bursa_Inf (L6_3 vs L7_2) represents the comparison between the line 6_3 and 7_2 MDV-infected birds.

Table S3. The canonical pathways and p value predicted with Ingenuity Pathway Analysis (IPA) analysis in the spleen using the differentially expressed MitoProteome genes.

No.	Canonical Pathway	Spleen_L6$_3$ (Con vs Inf)	Spleen_L7$_2$ (Con vs Inf)	Spleen_Con (L6$_3$ vs L7$_2$)	Spleen_Inf (L6$_3$ vs L7$_2$)
1	Mitochondrial Dysfunction	3.01	17.17	2.82	23.71
2	Oxidative Phosphorylation	2.16	11.87	0.87	21.45
3	Sirtuin Signaling Pathway	0.54	9.65	2.16	15.56
4	Folate Polyglutamylation		7.47		5.14
5	Acetate Conversion to Acetyl-CoA	2.34	3.31	2.27	3.31
6	Glutaryl-CoA Degradation	1.74	3.41	1.67	3.42
7	TCA Cycle II (Eukaryotic)		4.20		5.66
8	Gluconeogenesis I	1.53	2.78		5.48
9	Fatty Acid β-oxidation I	1.44	1.46	3.08	3.71
10	Tryptophan Degradation III (Eukaryotic)	1.55	2.83	1.48	2.83
11	Oxidative Ethanol Degradation III	1.62	1.81	3.45	1.81
12	Folate Transformations I		4.22		4.22
13	Induction of Apoptosis by HIV1	1.17	3.62		3.63
14	Tetrahydrofolate Salvage from 5,10-methenyltetrahydrofolate		3.09		5.14
15	Ethanol Degradation IV	1.55	1.66	3.29	1.67
16	4-aminobutyrate Degradation I	2.46	3.61		1.57
17	tRNA Charging		4.57	1.29	1.31
18	Histidine Degradation III		2.65		4.40
19	Ethanol Degradation II	1.38	1.35	2.95	1.35
20	Glutamate Degradation III (via 4-aminobutyrate)	2.24	3.09		1.35
21	Ketolysis		4.06		2.45
22	Glycine Cleavage Complex		1.27	2.10	2.92
23	Choline Degradation I		1.74	2.57	1.74

24	Aspartate Degradation II		1.21		4.60
25	Valine Degradation I	1.69	3.26		0.82
26	Isoleucine Degradation I		3.59		2.16
27	Glycolysis I		2.78		2.78
28	Superpathway of Cholesterol Biosynthesis		1.57		3.94
29	Superpathway of Geranylgeranyldiphosphate Biosynthesis I (via Mevalonate)		1.99		3.34
30	UDP-N-acetyl-D-galactosamine Biosynthesis II		1.02	1.83	2.37
31	Ketogenesis		2.45		2.45
32	Lysine Degradation II		1.35	2.18	1.35
33	NRF2-mediated Oxidative Stress Response		1.99		2.63
34	Glutathione Redox Reactions I		1.70		2.88
35	Mevalonate Pathway I		2.22		2.22
36	β-alanine Degradation I	2.64	1.74		
37	Apoptosis Signaling	0.98	1.93		1.24
38	Vitamin-C Transport		2.04		2.04
39	Parkinson's Signaling		2.04		2.04
40	Glutathione-mediated Detoxification		1.49		2.56
41	Acetyl-CoA Biosynthesis I (Pyruvate Dehydrogenase Complex)		2.77		1.21
42	Ubiquinol-10 Biosynthesis (Eukaryotic)	1.66	1.89		
43	Trehalose Degradation II (Trehalase)			2.18	1.35
44	Cytotoxic T Lymphocyte-mediated Apoptosis of Target Cells	1.44	1.46		0.60
45	Pyrimidine Deoxyribonucleotides De Novo Biosynthesis I		1.73		1.74
46	Stearate Biosynthesis I (Animals)		1.21		2.13
47	Heme Biosynthesis from Uroporphyrinogen-III I				3.31
48	Fatty Acid β-oxidation III (Unsaturated, Odd Number)				3.31
49	Interferon Signaling	1.39	1.37		0.55
50	Acyl-CoA Hydrolysis		2.29		0.98
51	Aspartate Biosynthesis		1.57		1.57

52	Glutamate Degradation II		1.57		1.57
53	NADH Repair		1.57		1.57
54	Inosine-5'-phosphate Biosynthesis II		1.57		1.57
55	4-hydroxyproline Degradation I		1.57		1.57
56	Tetrahydrobiopterin Biosynthesis II		1.57		1.57
57	Tetrahydrobiopterin Biosynthesis I		1.57		1.57
58	TNFR1 Signaling		1.12		1.97
59	D-myo-inositol (3,4,5,6)-tetrakisphosphate Biosynthesis	0.81	0.42	1.80	
60	D-myo-inositol (1,4,5,6)-Tetrakisphosphate Biosynthesis	0.81	0.42	1.80	
61	GDP-glucose Biosynthesis			1.88	1.06
62	Glutathione Redox Reactions II		1.44		1.44
63	L-cysteine Degradation I		1.44		1.44
64	Ascorbate Recycling (Cytosolic)		1.44		1.44
65	Arsenate Detoxification I (Glutaredoxin)		1.44		1.44
66	3-phosphoinositide Degradation	0.78	0.37	1.72	
67	Glucose and Glucose-1-phosphate Degradation			1.83	1.02
68	Pregnenolone Biosynthesis	1.86	0.98		
69	D-myo-inositol-5-phosphate Metabolism	0.77	0.36	1.70	
70	Huntington's Disease Signaling		0.71		2.09
71	2-ketoglutarate Dehydrogenase Complex		1.35		1.35
72	Tyrosine Degradation I		1.35		1.35
73	Histidine Degradation VI	1.76	0.89		
74	Estrogen Biosynthesis	1.33		1.27	
75	Heme Biosynthesis II				2.55
76	3-phosphoinositide Biosynthesis	0.68	0.26	1.53	
77	Superpathway of Inositol Phosphate Compounds	0.62	0.44	1.40	
78	Tumoricidal Function of Hepatic Natural Killer Cells		1.70		0.71
79	Aryl Hydrocarbon Receptor Signaling			1.82	0.43
80	Lysine Degradation V			2.18	

81	Acetyl-CoA Biosynthesis III (from Citrate)		2.04		
82	Superpathway of Methionine Degradation		1.35		0.54
83	Iron homeostasis signaling pathway		0.45		1.44
84	NAD Phosphorylation and Dephosphorylation			1.76	
85	Bile Acid Biosynthesis, Neutral Pathway			1.76	
86	Glycine Biosynthesis I		1.74		
87	CDP-diacylglycerol Biosynthesis I		1.70		
88	Phosphatidylglycerol Biosynthesis II (Non-plastidic)		1.63		
89	Histamine Degradation			1.60	
90	Methylglyoxal Degradation I		1.57		
91	Fatty Acid α-oxidation			1.54	
92	Putrescine Degradation III			1.52	
93	Tryptophan Degradation X (Mammalian, via Tryptamine)			1.48	
94	Geranylgeranyldiphosphate Biosynthesis				1.44
95	Proline Biosynthesis I				1.44
96	Methylmalonyl Pathway		1.44		
97	Serine Biosynthesis				1.35
98	Trans, trans-farnesyl Diphosphate Biosynthesis				1.35
99	dTMP De Novo Biosynthesis		1.35		
100	2-oxobutanoate Degradation I		1.35		
101	Dopamine Degradation			1.31	

Spleen_L6$_3$ (Con vs Inf) represents the comparison between the MDV-infected and control birds in line 6$_3$.

Spleen _L7$_2$ (Con vs Inf) represents the comparison between the MDV-infected and control birds in line 7$_2$.

Spleen _Con (L63 vs L72) represents the comparison between the line 6$_3$ and 7$_2$ control birds.

Spleen _Inf (L63 vs L72) represents the comparison between the line 6$_3$ and 7$_2$ MDV-infected birds.
